# Effects of Hangeshashinto on Growth of Oral Microorganisms

**DOI:** 10.1155/2015/512947

**Published:** 2015-06-15

**Authors:** Haruka Fukamachi, Chinami Matsumoto, Yuji Omiya, Takafumi Arimoto, Hirobumi Morisaki, Hideo Kataoka, Miki Kadena, Takahiro Funatsu, Masato Fukutake, Yoshio Kase, Hirotaka Kuwata

**Affiliations:** ^1^Department of Oral Microbiology and Immunology, Showa University School of Dentistry, 1-5-8 Hatanodai, Shinagawa-ku, Tokyo 142-8555, Japan; ^2^Tsumura Research Laboratories, Tsumura & Co., 3586 Yoshiwara, Ami-machi, Inashiki-gun, Ibaraki 300-1192, Japan; ^3^Division of Dentistry for Persons with Disabilities, Department of Special Needs Dentistry, Showa University School of Dentistry, 2-1-1 Kitasenzoku, Ohta-ku, Tokyo 145-8515, Japan

## Abstract

Oral mucositis (OM) in cancer patients induced by chemotherapy or radiotherapy has a significant impact on quality of life, and causes considerable morbidity. Oral microorganisms are likely to intensify the inflammatory process and aggravate the formation of ulcers. Hangeshashinto (HST), a Japanese kampo medicine, has been reported to be effective when used as a gargle for the treatment of OM. To clarify the effects of HST on oral microorganisms, we assessed its antimicrobial activity against 27 microbial species, including 19 oral bacteria and one fungus. HST extract inhibited the growth of Gram-negative bacteria, including *Fusobacterium nucleatum, Porphyromonas gingivalis, Porphyromonas endodontalis, Prevotella intermedia, Prevotella melaninogenica, Tannerella forsythia, Treponema denticola,* and *Porphyromonas asaccharolytica,* though inhibitory effects were less pronounced for Gram-positive bacteria and the fungal strain. We then investigated the effects of antibacterial activities on 15 purified ingredients of HST and determined that baicalein, berberine, coptisine, [6]-shogaol, and homogentisic acid actively inhibited the growth of these bacteria. These findings showed that HST inhibits the growth of specific Gram-negative periodontopathogenic bacteria, which are significant pathogens in OM, without disturbing the normal oral flora. Our data suggest that HST may be a useful treatment for OM in patients undergoing anticancer treatment.

## 1. Introduction

Chemotherapy and radiotherapy produce side effects that have a major impact on the quality of life in patients while undergoing treatment. Oral mucositis (OM) is one of the most frequently encountered side effects and commonly occurs 7–10 days after therapy. Pain associated with OM can prevent patients from eating, which results in a poorer treatment outcome. The complications arising from severe OM frequently require a temporary or complete cessation of therapy, thus preventing application or completion of the planned treatment dose. To date, a reliable treatment for OM has not been established.

Recently, a biological model for OM was proposed by Sonis, which revealed the complexity of pathogenesis of this disease [1]. The model described four successive phases in the disease process, an inflammatory (connective tissue) phase, followed by an epithelial phase, which leads to an ulcerative/bacteriological phase, and finally a healing phase. Oral microorganisms are thought to be involved in the ulcerative/bacteriological phase, where they are likely to intensify the inflammatory process and aggravate or promote the formation of ulcers [1–3]. In addition, the presence of OM may act as a portal of entry for oral microorganisms into the blood stream, leading to bacteremia and sepsis. Recent studies reported a relationship between the prevalence of oral pathologic condition and the occurrence of OM in patients undergoing anticancer therapy [4, 5]. According to these reports, patients with gingivitis or periodontal disorders had high frequency of OM. In addition, Laheij et al. reported that there is a relationship between several periodontopathogenic bacteria, yeasts, and OM in patients being treated with high-dose chemotherapy for hematopoietic stem cell transplant [6]. Therefore, we hypothesized that periodontopathogenic bacteria may aggravate the inflammatory process in OM.

Hangeshashinto (HST) is one of the Japanese “*Kampo*” medicines. HST is frequently used to treat acute or chronic gastrointestinal catarrh, fermentative diarrhea, and acute enterogastritis, and the mechanisms of the pharmacological effects have been partially elucidated. Recently, clinical trials demonstrated that an HST gargle had therapeutic effects in patient with chemotherapy-induced OM [7] and (chemo-) radiation-induced OM [8]. In addition, the study revealed that HST inhibited the increase in prostaglandin E2 (PGE2) production from IL-1*β* stimulated-human oral keratinocytes [9]. HST is composed of seven crude drugs, namely, pinellia tuber, scutellaria root, glycyrrhiza, jujube, ginseng, coptis rhizome, and processed ginger, all of which have medicinal properties. Coptis rhizome also has strong wide-spectrum antibacterial activity [10–12]. Therefore, in addition to the suppression of PGE2 production, the therapeutic effects of HST might also arise from antibacterial activity against oral microorganisms. By reducing the impact of the oral microorganisms, especially periodontopathogenic bacteria, HST might help reduce cancer therapy-related complications, as outlined in the four-stage model of OM.

The aims of this study were to assess the antimicrobial activity of HST extract and that of the purified active ingredients. We examined the antimicrobial activity of HST extract on the growth of 27 microbes, including 19 oral bacteria and one fungus. Furthermore, we studied the antibacterial effects of 15 purified ingredients contained in HST.

## 2. Materials and Methods

### 2.1. Bacterial Strains and Culture Conditions

The bacterial strains examined in this study and their culture conditions are listed in Supplementary Table 1 (see Supplementary Material available online at http://dx.doi.org/10.1155/2015/512947).

### 2.2. HST

HST (lot number 2100014010), containing seven crude drugs (Pinellia tuber (tuber of* Pinellia ternata *BREITENBACH, Araceae), scutellaria root (root of* Scutellaria baicalensis* GEORGI, Labiatae), glycyrrhiza (root or stolon of* Glycyrrhiza uralensis *FISCHER, Leguminosae), jujube (fruit of* Zizyphus jujuba *Miller var.* inermis *REHDER, Rhamnaceae), ginseng (root of* Panax ginseng* C.A. MEYER, Araliaceae), coptis rhizome (rhizome of* Coptis japonica* MAKINO, Ranunculaceae), and processed ginger (steamed rhizome of* Zingiber officinale* ROSCOE, Zingiberaceae)), was obtained from Tsumura & Co. (Tokyo, Japan).

### 2.3. Preparation of HST Extract

HST was suspended in deionized water at 20 mg/mL and then stirred for 2 h. The suspension was autoclaved at 121°C for 20 min. After mixing for 10 min, the suspension was centrifuged at 3,500 ×g for 10 min. The supernatant was collected for use as the stock solution and stored at −20°C until use. The test solution was diluted to 10 mg/mL using culture medium.

### 2.4. Purified Ingredients from HST Extract

Fifteen purified active ingredients from HST were purchased from Tsumura & Co. (Supplementary Table 2). Stock solutions (30 mM) were prepared for each chemical in 100% dimethyl sulfoxide (DMSO) and stored at −80°C until use.

### 2.5. Minimum Inhibitory Concentration (MIC) and Minimum Bactericidal Concentration (MBC) Assays for HST

The MIC of HST against all 27 microbial species was determined using a 96-well plate dilution method. The 10 mg/mL test solution was prepared by two-fold serial dilution in each culture medium. Five concentrations were used within the 0.625–10 mg/mL range. The final inoculum concentrations were 1 × 10^4^ colony-forming units (CFU)/mL for Gram-positive bacteria and the fungal strain and 1 × 10^5^ CFU/mL for Gram-negative bacteria. The 96-well plates were incubated at 37°C for 24 h for Gram-positive bacteria and 48 h for Gram-negative bacteria. Medium without HST was used as a positive control. The MIC was defined as the lowest concentration that yielded no visible growth. The MBC of the test solution was determined by inoculating agar plates with the test solution mixture at a concentration representing the MIC, as well as at the next two higher concentrations. Experiments were performed in triplicate.

### 2.6. MIC and MBC Assays for 15 Purified Ingredients

The MIC of the 15 purified ingredients against* F. nucleatum, Por. gingivalis, Por. endodontalis, Pre. intermedia, Pre. melaninogenica, Tan. forsythia, Tre. denticola,* and* Por. asaccharolytica* was determined using the 96-well plate dilution method described above. Stock solutions (30 mM) were diluted with each culture medium to prepare test solutions at concentrations of 100 *μ*M, 200 *μ*M, and 300 *μ*M. The 100 *μ*M test solutions were prepared by twofold serial dilution using each culture medium. Seven concentrations were used within the 1.56–100 *μ*M range at final DMSO concentrations of 0.005–0.33%. In case of* Tan. forsythia*, additional test solutions were used at concentrations of 200 *μ*M and 300 *μ*M, with final DMSO concentrations of 0.67% and 1%, respectively. The concentration of DMSO was less than 1% and thus did not affect the microbial growth. Wells containing 100 *μ*L of each dilution were inoculated with bacteria. The final inoculum concentrations were 1 × 10^5^ CFU/mL. The 96-well plates were incubated at 37°C for 48 h. Medium containing 0.33%, 0.67%, and 1% DMSO was used as a control. The MIC was defined as the lowest antibiotic concentration that yielded no visible growth. The MBC of the test solution was determined using a method similar to that described above. Experiments were performed in at least quadruplicate.

### 2.7. Time-Kill Analysis of HST for* Porphyromonas gingivalis*

The time-kill analysis was determined as follows. Sample solutions, including culture broth and HST extract at a final concentration of 10 mg/mL, were inoculated with bacterial suspension in triplicate. The final inoculum concentrations were 1 × 10^5^ CFU/mL. Controls containing no HST extract were also included. The number of viable bacterial cells was determined at 5, 10, 15, 60, and 120 min after inoculation by plating aliquots of undiluted and 10-fold serially diluted sample onto BHI H/M plates. Plates were incubated for 72 h at 37°C, and the resultant colonies were counted. Data from triplicate assays were averaged and plotted for each time point. All experimentation was carried out under anaerobic conditions (10% CO_2_, 10% H_2_, and 80% N_2_).

## 3. Results

### 3.1. The Antibacterial Effect of HST

To determine whether the HST extract could inhibit the growth of oral microorganisms, we evaluated the antibacterial effect of HST against 27 microorganisms, including 19 oral bacteria and one fungus. A clear difference in susceptibility among species was observed (Table 1). The specific Gram-negative periodontopathogenic bacteria, including* F. nucleatum*,* Por. gingivalis*,* Por. endodontalis*,* Pre. intermedia*,* Pre. melaninogenica*,* Tan. forsythia*,* Tre. denticola*, and* Por. asaccharolytica*, were susceptible to HST extract and had low MICs (<5 mg/mL). However, the inhibitory effects of HST on Gram-positive bacteria and fungus were much less pronounced. The MICs and MBCs of HST extract are shown in Table 1.

### 3.2. The Antibacterial Effects of 15 Ingredients Contained in HST Extract

To identify the antimicrobial components of HST extract, the susceptibilities of* F. nucleatum, Por. gingivalis, Por. endodontalis, Pre. intermedia, Pre. melaninogenica, Tan. forsythia, Tre. denticola*, and* Por. asaccharolytica* to 15 ingredients present in HST extract were examined.  Table 2 summarizes the antibacterial activity of these ingredients. From this analysis, baicalein, berberine, coptisine, [6]-shogaol, and homogentisic acid were found to have antibacterial activity, whereas 10 other ingredients, including baicalin, wogonin, acteoside, [6]-gingerol, liquiritin, glycyrrhizic acid, ginsenoside Rg1, ginsenoside Rb1, corymboside, and cyclic AMP exerted no inhibitory effect on any of the strains. The MICs and MBCs of active ingredients are given in Table 2.

### 3.3. Time-Kill Analysis of HST against* Por. gingivalis*

**Figure 1:**
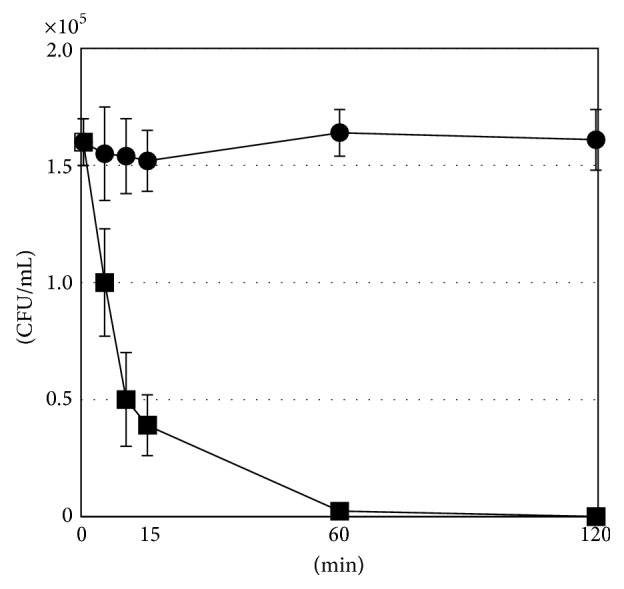
Time-kill curve of HST extract against* Porphyromonas gingivalis* ATCC33277. Data points represent the viable cell numbers in culture medium (●) or culture medium containing 10 mg/mL HST (■). The experiment was performed in triplicate and data points are the means of 6 replicates ± SD.

The kinetics of the antibacterial activity of HST against the typical periodontopathogenic bacterium,* Por. gingivalis* ATCC33277, were examined. A representative time-kill profile is shown in Figure 1. In treatments at the MIC (2.5 mg/mL), the number of viable cells did not decrease until 120 min after inoculation, and bacterial death was observed at 6 h after inoculation (data not shown). However, when the concentration reached four times the MIC (10 mg/mL), the number of viable cells decreased by approximately 40% after 5 min, 98% after 60 min, and 100% after 120 min after inoculation.

## 4. Discussion

The aim of this study was to clarify the effects of HST on the survival of oral microorganisms. We first assessed the antimicrobial activity of HST extract against 27 microbial species, including 19 oral bacteria and one fungus. HST extract strongly inhibited the growth of Gram-negative periodontopathogenic bacteria such as* F. nucleatum*,* Por. gingivalis*,* Por. endodontalis*,* Pre. intermedia*,* Pre. melaninogenica, Tan. forsythia*,* Tre. denticola*, and* Por. asaccharolytica*, though it was less effective against Gram-positive bacteria and fungi. Donnelly et al. reported that, in cancer patients, the oral microbial balance can be disturbed by the cancer itself, the anticancer treatment, or the supportive therapies. All of these factors may contribute to a shift in the make-up of the microflora of the oral cavity from mainly Gram-positive to predominantly Gram-negative bacteria [3]. The cell wall of Gram-negative bacteria contains the endotoxin lipopolysaccharide (LPS). LPS activates macrophages to produce inflammatory mediators. The disruption of the oral microbial balance may intensify the inflammatory process and aggravate or promote the formation of ulcers. Our present results indicated that HST extract might be useful for treatment of OM, as it showed antibacterial activity specific to Gram-negative bacteria. Furthermore, these results indicated that HST may also be useful for treatment of periodontal disease.

HST is composed of seven crude drugs, including pinellia tuber, scutellaria root, glycyrrhiza, jujube, ginseng, coptis rhizome, and processed ginger, all of which contain crude drugs. A previous three-dimensional high-performance liquid chromatography analysis determined the broad chemical profile of HST. At least 30 ingredients were identified in the chromatogram (data not shown). We tested the antibacterial activity of 15 major ingredients of HST (baicalin, baicalein, wogonin, berberine, coptisine, [6]-shogaol, [6]-gingerol, liquiritin, glycyrrhizic acid, acteoside, ginsenoside Rg1, ginsenoside Rb1, corymboside, homogentisic acid, and cyclic AMP) towards* F. nucleatum*,* Por. gingivalis*,* Por. endodontalis*,* Pre. intermedia*,* Pre. melaninogenica, Tan. forsythia*,* Tre. denticola*, and* Por. asaccharolytica*, all of which were susceptible to HST extract. Of the 15 compounds, baicalein, berberine, coptisine, [6]-shogaol, and homogentisic acid showed antibacterial activity, although the activity differed between the bacterial species.

Isoquinoline alkaloid, berberine, and coptisine were highly active against* F. nucleatum*,* Por. gingivalis*,* Por. endodontalis*,* Pre. intermedia*,* Pre. melaninogenica*,* Tre. denticola*, and* Por. asaccharolytica*, though no inhibitory effect was observed on the growth of* Tan. forsythia*. Species-specific inhibition by berberine has been reported previously [11, 13]. According to these reports,* Por. gingivalis*,* Pre. intermedia, *and* F. nucleatum* were susceptible to berberine at an MIC range of 13–50 *μ*g/mL (35–134 *μ*M), which agrees with the findings of the current study. In addition, our study revealed that several Gram-negative periodontopathogenic bacteria, including* Por. endodontalis*,* Pre. melaninogenica, *and* Tre. denticola*, are susceptible to berberine at a similar MIC range. HST extract also contained the alkaloid coptisine, the inhibitory activity of which was approximately equal to that of berberine. Except for* Tre. denticola*, the MBC values were in the same range as those of the MIC values for all microbial species, indicating that berberine and coptisine likely have bactericidal activity.

Interestingly, [6]-shogaol exhibited species-specific antibacterial activity against the* Prevotella *and* Porphyromonas* strains, while [6]-gingerol did not inhibit the growth of these species. The major active ingredients of processed ginger are gingerol and shogaol. Processed ginger has received extensive attention due to its antioxidant and anti-inflammatory activities [14]. Thermal processing of gingerols gives shogaols, which frequently show greater anticancer activity than their precursors [15, 16]. Similarly, in the current study, [6]-shogaol more effectively inhibited bacterial growth than [6]-gingerol. Despite this, Park et al. reported that [10]-, [12]-gingerol could inhibit the growth of* Por. gingivalis*,* Por. endodontalis*, and* Pre. intermedia* at an MIC range of 6–30 *μ*g/mL (21–107 *μ*M) and an MBC range of 4–20 *μ*g/mL (14–71 *μ*M) [17].

Baicalein is the common name for 5,6,7-trihydroxyflavone. In the current study, baicalein showed antibacterial activity towards all* Porphyromonas* and* Prevotella *species, except* Por. gingivalis*, and was the only compound with activity against* Tan. forsythia*. Baicalein is an aglycone of baicalin, but baicalin did not inhibit the growth of any of the tested bacteria.

Homogentisic acid is a phenolic acid. Recently, Lebouvier et al. reported that homogentisic acid has antiplasmodial activity, mediated through inhibition of Pfnek-1, which is a specific protein kinase of* Plasmodium falciparum* [18]. However, little is known about the antibacterial activity. For all of the ingredients identified here, the mechanisms of antibacterial activity remain unknown.

Kono et al. reported that the concentrations of baicalein, berberine, and [6]-shogaol in 10 mg/mL HST extracts were 8.56 *μ*g/mL (32 *μ*M), 37.59 *μ*g/mL (112 *μ*M), and 1.63 *μ*g/mL (5.9 *μ*M), respectively [9]. This suggests that the antibacterial activity of HST extract can be mainly attributed to berberine. In our study,* Por. gingivalis*,* Por. endodontalis*,* Por. asaccharolytica*,* Pre. intermedia*,* Pre. melaninogenica*,* Tre. denticola*, and* F. nucleatum* were susceptible to berberine at an MIC range of 25–100 *μ*M. Except for* Tre. denticola*, the MBC for berberine-susceptible bacteria was in the range of 25–100 *μ*M. This indicates that the antibacterial activity of berberine is effectively bactericidal. The MIC of baicalein for* Tan. forsythia *was 300 *μ*M, whereas the baicalein concentration in HST extract was only 32 *μ*M. These results raise the possibility that other antibacterial ingredients are present in HST or that an antibacterial effect is provided by the combination of ingredients.

A cell viability assay using human oral keratinocytes demonstrated that HST extract may not induce a cytotoxic effect at 10 mg/mL at 30 min after inoculation but that cell viability was significantly affected by the 60 min time point (Supplementary Figure 1). On the other hand, the survival of* Por. gingivalis* was dramatically decreased at HST concentrations greater than four times the MIC at 15 min after inoculation. After a patient with OM has rinsed with HST, a gradual decrease in HST concentration may occur in the oral cavity due to dilution by saliva. Further studies are needed to determine a HST concentration that may inhibit bacterial growth without affecting the survival of oral cells* in vivo*. In addition, the oral cavity is heavily colonized by a complex, relatively specific, and highly interrelated group of microorganisms that are organized in biofilms. Biofilms can be more resistant to antimicrobials than planktonic cells from the same species. Further studies should evaluate whether the antibacterial activity of HST is maintained against bacteria present in a biofilm.

The complexity of the pathogenesis of OM in cancer patients has been revealed. However, interventions that target only one aspect of the OM biological phase have been reported to be largely ineffective [19]. Treatments of OM in cancer patients should be directed toward multiple biological targets of OM process. Kono et al. reported that HST strongly suppresses PGE2. The present study showed that HST exhibited antibacterial activity against Gram-negative periodontopathogenic bacteria and that ingredients in HST extract may play a role in inhibiting the growth of oral bacteria. Although the mechanism of inhibition of bacterial growth is not fully understood, the data from this investigation indicated a potential application for HST as a therapeutic agent for the prevention of the Gram-negative periodontopathogenic bacterial infection in OM.

## Supplementary Material

Supplementary Table 1 presents the bacterial strains and their culture conditions.
Supplementary Table 2 shows seven constituent crude drugs of HST and origin of fifteen purified compounds.
Supplementary Figure 1 shows the effect of HST extract on cell viability of human oral keratinocytes.

## Figures and Tables

**Table 1:** Minimal inhibitory concentrations (MIC) and minimal bactericidal concentrations (MBC) of HST extract.

Bacterial strain	MIC	MBC
	(mg/mL)	(mg/mL)
Gram-negative oral bacteria		
*Aggregatibacter actinomycetemcomitans* Y4	>10	NT^*∗*^
*Campylobacter rectus* ATCC33238	>10	NT^*∗*^
*Capnocytophaga ochracea* ATCC27282	>10	NT^*∗*^
*Fusobacterium nucleatum* ATCC25586	1.25	1.25
*Porphyromonas gingivalis* ATCC33277	2.5	2.5
*Porphyromonas gingivalis* 381	1.25	1.25
*Porphyromonas endodontalis* ATCC35406	<0.625	<0.625
*Prevotella intermedia* ATCC25611	2.5	2.5
*Prevotella melaninogenica* ATCC25845	2.5	2.5
*Tannerella forsythia* ATCC43037	5.0	ND^*∗∗*^
*Treponema denticola* ATCC35405	5.0	ND^*∗∗*^
*Veillonella parvula* ATCC17745	>10	NT^*∗*^
Gram-negative bacteria		
*Bacteroides thetaiotaomicron* JCM5827	>10	NT^*∗*^
*Campylobacter jejuni* ATCC29428	10	10
*Escherichia coli* K12	>10	NT^*∗*^
*Porphyromonas asaccharolytica* JCM6326	2.5	2.5
Gram-positive oral bacteria		
*Actinomyces viscosus* ATCC19246	>10	NT^*∗*^
*Enterococcus faecalis* 4532D	>10	NT^*∗*^
*Lactobacillus casei* JCM1133	>10	NT^*∗*^
*Streptococcus anginosus* IS57	>10	NT^*∗*^
*Streptococcus gordonii* ATCC10558	>10	NT^*∗*^
*Streptococcus mutans* 109c	>10	NT^*∗*^
*Streptococcus salivarius* JCM5707	>10	NT^*∗*^
Gram-positive bacteria		
*Bacillus subtilis* ATCC9372	>10	NT^*∗*^
*Bifidobacterium longum* JCM1222	10	ND^*∗∗*^
*Staphylococcus aureus* ATCC6738	>10	NT^*∗*^
*Streptococcus pyogenes* ATCC12348	>10	NT^*∗*^
Fungus		
*Candida albicans* JCM2085	>10	NT^*∗*^

NT^*∗*^: not tested; ND^*∗∗*^: not detected.

**Table 2:** Minimal inhibitory concentrations (MIC) and minimal bactericidal concentrations (MBC) of 15 purified compounds.

	MIC/MBC (*μ*M)								
	1	2	3	4	5	6	7	8	9
Baicalin	—	—	—	—	—	—	—	—	—
Baicalein	—	—	—	25/25	100/100	25/ND	300/300	—	25/25
Wogonin	—	—	—	—	—	—	—	—	—
Acteoside	—	—	—	—	—	—	—	—	—
Berberine	100/100	25/50	50/50	100/100	25/25	100/100	—	25/ND	100/100
Coptisine	50/50	25/50	50/50	100/100	25/25	50/100	—	12.5/ND	100/100
[6]-Shogaol	—	50/50	25/50	25/25	100/100	50/100	—	—	12.5/12.5
[6]-Gingerol	—	—	—	—	—	—	—	—	—
Liquiritin	—	—	—	—	—	—	—	—	—
Glycyrrhizic acid	—	—	—	—	—	—	—	—	—
Ginsenoside Rg1	—	—	—	—	—	—	—	—	—
Ginsenoside Rb1	—	—	—	—	—	—	—	—	—
Corymboside	—	—	—	—	—	—	—	—	—
Homogentisic acid	—	—	—	—	25/25	—	—	—	12.5/12.5
Cyclic AMP	—	—	—	—	—	—	—	—	—

—: not active; ND: not detected.

1: *F*. *nucleatum*; 2: *P*. *gingivalis* ATCC33277; 3: *P*. *gingivalis* 381; 4: *P*. *endodontalis*; 5: *P*. *intermedia*; 6: *P*. *melaninogenica*; 7: *T*. *forsythia*; 8: *T*. *denticola*; 9: *P*. *asaccharolytica*.
