# Supplementary material for: Effects of Hangeshashinto on Growth of Oral Microorganisms
Source: Evid Based Complement Alternat Med. 2015 Jun 15;2015:512947. doi: 10.1155/2015/512947 (PMC4485921; doi:10.1155/2015/512947)
Supplement: Supplementary file 1 — Supplementary Table 1 presents the bacterial strains and their culture conditions. Supplementary Table 2 shows seven constituent crude drugs of HST and origin of fifteen purified compounds. Supplementary Figure 1 shows the effect of HST extract on cell viability of human oral keratinocytes. [file 512947.f1.pdf]

Supplementary Table 1 Bacterial strains and culture conditions

Bacterial strain	Culture medium	Culture condition
**Gram-negative Oral Bacteria**		
Aggregatibacter actinomycetemcomitans Y4	BHI H/M	Capneic
Camphylobacter rectus ATCC33238	BHI NaCl	Anaerobic
Capnocytophaga ochracea ATCC27282	BHI H/M	Anaerobic
*Fusobacterium nucleatum*ATCC25586	BHI H/M	Anaerobic
Porphyromonas gingivalis ATCC33277	BHI H/M	Anaerobic
Porphyromonas gingivalis 381	BHI H/M	Anaerobic
Porphyromonas endodontalis ATCC35406	BHI H/M	Anaerobic
Prevotella intermedia ATCC25611	BHI H/M	Anaerobic
Prevotella melaninogenica ATCC25845	BHI H/M	Anaerobic
Tannerella forsythia ATCC43037	TF medium	Anaerobic
Treponema denticola ATCC35405	GM-1	Anaerobic
Veillonella parvula ATCC17745	BHI H/M	Microaerobic
**Gram-negative Bacteria**		
Bacteroides thetaiotaomicron JCM5827	BHI H/M	Anaerobic
Camphylobacter jejuni ATCC29428	BHI NaCl	Microaerobic
Escherichia coli K12	THB	Aerobic
Porphyromonas asaccharolytica JCM6326	BHI H/M	Anaerobic
**Gram-positive Oral Bacteria**		
Actinomyces viscosus ATCC19246	THB	Capneic
Enterococcus faecalis 4532D	THB	Anaerobic
Lactobacillus casei JCM1133	THB	Anaerobic
Streptococcus anginosus IS57	THB	Anaerobic
Streptococcus gordonii ATCC10558	THB	Anaerobic
Streptococcus mutans 109c	THB	Anaerobic
Streptococcus salivarius JCM5707	THB	Anaerobic
**Gram-positive Bacteria**		
Bacillus subtilis ATCC9372	BHI H/M	Aerobic
Bifidobacterium longum JCM1222	THB	Anaerobic
Staphyrococcus aureus ATCC6738	THB	Aerobic
Streptococcus pyogenes ATCC12348	THB	Anaerobic
**Fungus**		
Candida albicans JCM2085	BHI	Aerobic

BHI H/M: brain-heart infusion broth with hemin (5 µg/ml) and menadione (5 µg/ml); GM-1: as described previously (Blakemore, 1976); TF medium: as described previously (Shimotahira, 2013); BHI NaCl: BHI containing 0.3% NaCl; THB: Todd Hewitt Broth. The anaerobic organisms were cultured anaerobically using an AnaeroPack-Anaero system (Mitsubishi Gas Chemical Company) at 37°C. The microaerobic organisms were cultured microaerobically using an AnaeroPack-MicroAero system at 37°C. The capneic organisms were cultured capneically using an AnaeroPack-CO2 system at 37°C.

Supplementary Table 2 List of crude drugs and their purified extracts

Crude drugs	Purified compound	Reference
Scutellariae radix	Baicalin	Kono, 2014
	Baicalein	Kono, 2014
	Wogonin	Kono, 2014
	Acteoside	Xie, 2002
Coptidis rhizome	Berberine chloride	Kono, 2014
	Coptisine	Kono, 2014
Zingiberis siccatum rhizoma	[6]-Shogaol	Kono, 2014
	[6]-Gingerol	Kono, 2014
Glycyrrhizae radix	Liquiritin	Kono, 2014
	Glycyrrhizic acid	Kono, 2014
Ginseng radix	Ginsenoside Rg1	Qian, 2009
	Ginsenoside Rb1	Qian, 2009
Pinelliae tuber	Corymboside	Wagner, 2011
	Homogentisic acid	Hatano, 1991
Zizyphi fructus	Cyclic AMP	Hanabusa, 1981

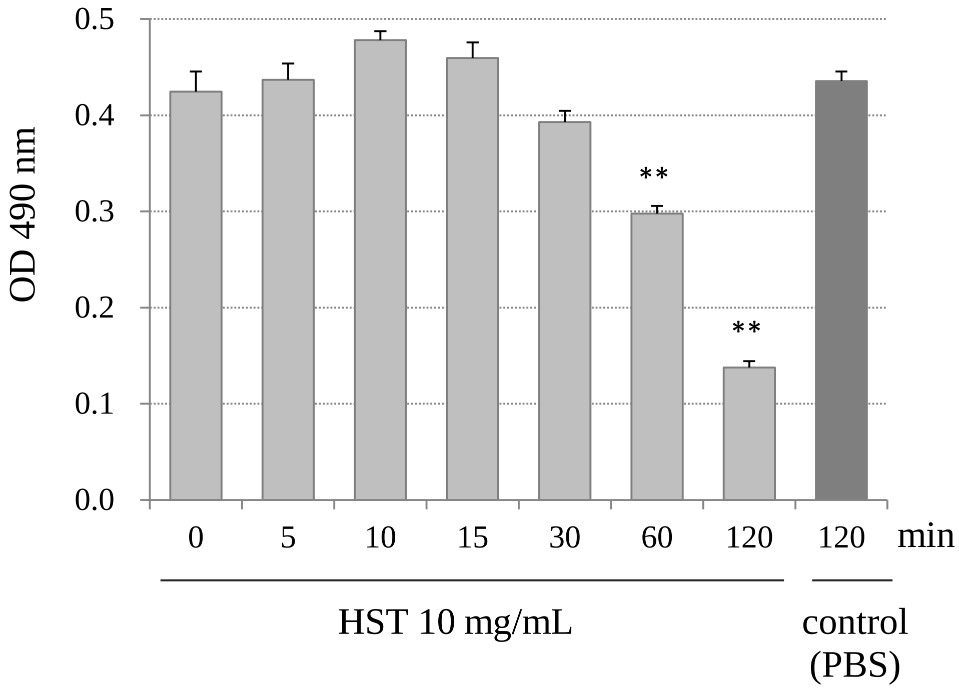

Supplementary Figure 1 Viability of human oral keratinocytes. We seeded 5×10^{3} cells in 96-well plates and exposed them to 10 mg/ml HST extract for 0, 5, 10, 15, 30, 60, and 120 min. Cell viability was estimated by measuring MTS. Graph shows mean absorbance at 490 nm for the detection of MTS readings from a microplate reader. Error bars correspond to standard error. **P<0.01 vs. control by Dunnett's test.

References

- Blakemore, R. P., E. Canale-Parola (1976). Arginine catabolism by *Treponema denticola*. *J bacteriology* 128:616-622.

- Shimotahira N, Oogai Y, Kawada-Matsuo M, Yamada S, Fukutsuji K, Nagano K, Yoshimura F, Noguchi K, Komatsuzawa H (2013). The surface layer of *Tannerella forsythia* contributes to serum resistance and oral bacterial coaggregation. *Infect Immun*. 81:1198-206.

- Xie LH, Wang X, Basnet P, Matsunaga N, Yamaji S, Yang DY, Cai SQ, Tani T (2002). Evaluation of variation of acteoside and three major flavonoids in wild and cultivated Scutellaria baicalensis roots by micellar electrokinetic chromatography. *Chem Pharm Bull*. 50:896-9.

- Qian ZM, Lu J, Gao QP, Li SP (2009). Rapid method for simultaneous determination of flavonoid, saponins and polyacetylenes in folium ginseng and radix ginseng by pressurized liquid extraction and high-performance liquid chromatography coupled with diode array detection and mass spectrometry. *J Chromatogr A*. 1216:3825-30.

- Wagner H, Bauer R, Melchart D, Xiao PG, Staudinger A (2011). Rhizoma Pinelliae — Banxia : *Chromatographic Fingerprint Analysis of Herbal Medicines*, Springer Vienna, pp 71-81 DOI: 10.1007/978-3-7091-0763-8_7

- Hanabusa K, Cyong J, Takahashi M (1981). High-level of cyclic AMP in the jujube plum. *Planta Med*. 42:380-4.

- Hatano K, Shoyama Y, Nishioka I (1991). Cultivation studies on Clonally Propagated Pinellia ternata. *Shoyakugaku Zasshi* 45:203-205.

- Kono T, Kaneko A, Matsumoto C, Miyagi C, Ohbuchi K, Omiya Y, Mizuhara Y, Miyamo K, Uezono Y (2014). Multitargeted effect of hangeshashinto for treatment of chemotherapy-inducd oral mucositis on inducible PGE2 production in human oral keratinocytes. *Integr Cancer Ther.* 13:435-45.
